# Supplementary material for: Identification of Freezing-Responsive microRNAs and Their Targets in Chinese Jujube by Small RNA and Degradome Sequencing
Source: Int J Mol Sci. 2026 Sep 4;27(17):7912. doi: 10.3390/ijms27177912 (PMC13566890; doi:10.3390/ijms27177912)
Supplement: Supplementary file 1 [file ijms-27-07912-s001.zip › Supplementary Material S2/novel_miR_196.pdf]

Provisional ID : novel_miR_196
Score total : 900.9
Score for star read(s) : 3.9
Score for read counts : 894.4
Score for mfe : 1
Score for randfold : 1.6
Score for cons. seed :
Total read count : 1766
Mature read count : 1748
Loop read count : 0
Star read count : 18

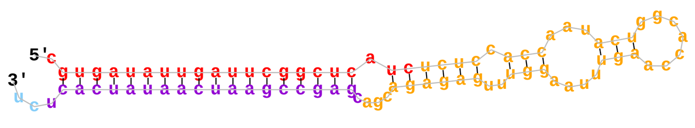

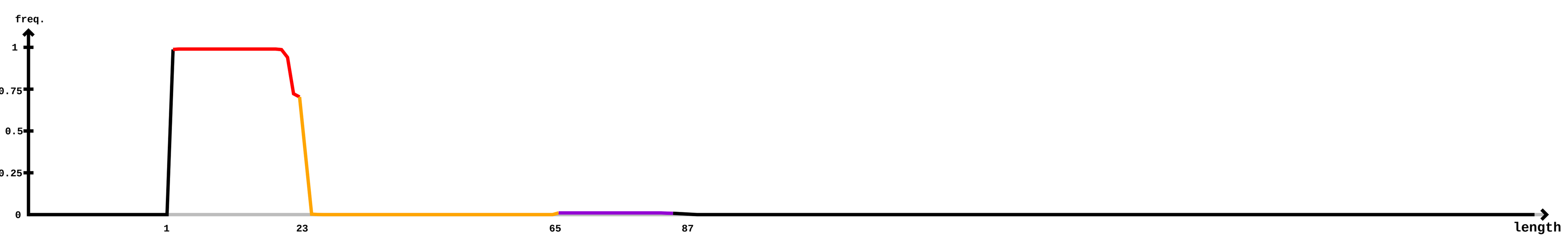

Mature Star

```
5'- cauucugcagaaguagacauggcgugauauugauucggcucaucucuccaccaauacuggcaccaaguuuaagguuugagagacgacgagccgaaucaauaucacucuuguaugcuucuucgcauauuaucaagguuuuuccuguuuuuugaguuccaauauuagccaagaagcaucucugcaggcaacucaugcauuaagcuacauagaagcuuguuuuggcucuccaucccccucuuguaaagucuuc -3' obs
    cauucugcagaaguagacauggcgugauauugauucggcucaucucuccaccaauacuggcaccaaguuuaagguuugagagacgacgagccgaaucaauaucacucuuguaugcuucuucgcauauuaucaagguuuuuccuguuuuuugaguuccaauauuagccaagaagcaucucugcaggcaacucaugcauuaagcuacauagaagcuuguuuuggcucuccaucccccucuuguaaagucuuc     exp
    ....((((((........((.((((((((((((((((.((((((.(((...(((......)))....)))..))))))....))))))))))))))))).)).((((((.....))))))......((.....))((((((((((.(((.......))))))))))))..)))))))(((.....)))..((((((......))))))....((((...((........))...))))...   reads  mm  sample
    ......................cgugauauugauucggcu..........................................................................................................................................................................................   5    0   LT-3
    ......................cgugauauugauucggcu..........................................................................................................................................................................................   1    0   LT-1
    ......................cgugauauugauucggcuc.........................................................................................................................................................................................   7    0   LT-1
    ......................cgugauauugauucggcuc.........................................................................................................................................................................................   13   0   LT-3
    ......................cgugauauugauucggcuU.........................................................................................................................................................................................   1    1   LT-3
    ......................cgugauauugauucggcuc.........................................................................................................................................................................................   33   0   LCK-3
    ......................cgugauauugauucggcuc.........................................................................................................................................................................................   21   0   LT-2
    ......................cgugCuauugauucggcuc.........................................................................................................................................................................................   1    1   LCK-3
    ......................cgugauauugauucggcuA.........................................................................................................................................................................................   6    1   LCK-3
    ......................cgugauCuugauucggcuca........................................................................................................................................................................................   1    1   LCK-2
    ......................cgugauCuugauucggcuca........................................................................................................................................................................................   1    1   LT-3
    ......................cgugauauugauucggcuca........................................................................................................................................................................................   60   0   LCK-2
    ......................cgugauauugauucggcuca........................................................................................................................................................................................   103  0   LCK-3
    ......................cgugauauugauucggcuca........................................................................................................................................................................................   23   0   LT-1
    ......................cgugauauugauucggcuca........................................................................................................................................................................................   3    0   JCK-3
    ......................cgugauauugauucggcuca........................................................................................................................................................................................   52   0   LT-3
    ......................cgugauauugauucgAcuca........................................................................................................................................................................................   1    1   LCK-3
    ......................cgugauauugauucggcuca........................................................................................................................................................................................   3    0   JT-3
    ......................cgugauauugauucggcuca........................................................................................................................................................................................   9    0   JCK-2
    ......................cgugauauugauucggcuca........................................................................................................................................................................................   1    0   JT-2
    ......................cgugauCuugauucggcuca........................................................................................................................................................................................   1    1   LCK-1
    ......................cgugauauugauucggcuca........................................................................................................................................................................................   71   0   LT-2
    ......................cgugauauugauucggcuca........................................................................................................................................................................................   2    0   JCK-1
    ......................cgugauauugauucggcuca........................................................................................................................................................................................   48   0   LCK-1
    ......................cAugauauugauucggcuca........................................................................................................................................................................................   1    1   LT-2
    ......................cgugauauugauucggcucU........................................................................................................................................................................................   3    1   LT-2
    ......................cgugauCuugauucggcucau.......................................................................................................................................................................................   1    1   LT-3
    ......................cgugauauugauucggcucau.......................................................................................................................................................................................   8    0   LT-1
    ......................cgugauauugauucggcucau.......................................................................................................................................................................................   14   0   LCK-3
    ......................cgugauauugauucggcucaA.......................................................................................................................................................................................   1    1   LT-3
    ......................cgugauauugauucggcucau.......................................................................................................................................................................................   1    0   LT-3
    ......................cgugauauugauucggcucau.......................................................................................................................................................................................   9    0   LT-2
    ......................cNugauauugauucggcucauc......................................................................................................................................................................................   1    1   JCK-1
```

```
                      Mature                                                          Star
cauucugcagaaguagacauggcgugauauugauucggcucaucucuccaccaauacuggcaccaaguuuaagguuugagagacgacgagccgaaucaauaucacucuuguaugcuucuucgcauauuaucaagguuuuuccuguuuuuugaguuccaauauuagccaagaagcaucucugcaggcaacucaugcauuaagcuacauagaagcuuguuuuggcucuccauccccccucuuguaaagucuuc
......................cgugauauugauucggcucauc...............................................................................................................................................................................................   122   0   LT-2
......................cgugauauugauucggcucauU...............................................................................................................................................................................................   3     1   LT-1
......................cgugauauugauucggcucauU...............................................................................................................................................................................................   13    1   LT-2
......................cgugCuauugauucggcucauc...............................................................................................................................................................................................   1     1   LT-3
......................cguUauauugauucggcucauc...............................................................................................................................................................................................   1     1   LT-2
......................cgugauauugauucggcCcauc...............................................................................................................................................................................................   1     1   LT-1
......................cgugauauugauucggcucauU...............................................................................................................................................................................................   5     1   JCK-3
......................cgugauauugauucggcucauc...............................................................................................................................................................................................   93    0   JCK-3
......................cgugauauugauucggcucauU...............................................................................................................................................................................................   5     1   LT-3
......................cgugauauugCuucggcucauc...............................................................................................................................................................................................   1     1   JCK-1
......................cgGgauauugauucggcucauc...............................................................................................................................................................................................   1     1   LT-2
......................cgugauauugCuucggcucauc...............................................................................................................................................................................................   1     1   LT-1
......................cgugauauugauucggcucauc...............................................................................................................................................................................................   237   0   LT-1
......................cgugauauugauucggcucauc...............................................................................................................................................................................................   31    0   LCK-1
......................cgugauauugauucggcucauc...............................................................................................................................................................................................   51    0   JCK-1
......................cgugauauugauucggcucauc...............................................................................................................................................................................................   99    0   JCK-2
......................cgugauauugauucggcucauc...............................................................................................................................................................................................   248   0   LT-3
......................cgugCuauugauucggcucauc...............................................................................................................................................................................................   1     1   LCK-3
......................cgugauauugCuucggcucauc...............................................................................................................................................................................................   1     1   JCK-2
......................cgugauauugauucggcucauU...............................................................................................................................................................................................   1     1   JCK-1
......................cgugauauugauucggcucauU...............................................................................................................................................................................................   9     1   LCK-3
......................cgugauauugauucggcucauc...............................................................................................................................................................................................   31    0   LCK-2
......................cgugauauugauucggcucauU...............................................................................................................................................................................................   1     1   JCK-2
......................Agugauauugauucggcucauc...............................................................................................................................................................................................   1     1   LT-1
......................cguAauauugauucggcucauc...............................................................................................................................................................................................   1     1   JCK-3
......................cgugauauugauucggcucCuc...............................................................................................................................................................................................   1     1   LT-2
......................cgugCuauugauucggcucauc...............................................................................................................................................................................................   2     1   JCK-2
......................cgugauauugauucggcucauc...............................................................................................................................................................................................   137   0   LCK-3
......................cgugauauugauucggcucaucA..............................................................................................................................................................................................   2     1   LT-1
......................cgugauauugauucggcucaucu..............................................................................................................................................................................................   34    0   LT-3
......................cgugauauugauucggcucaucu..............................................................................................................................................................................................   15    0   LCK-3
......................cgugauauugauucggcucaucu..............................................................................................................................................................................................   4     0   JCK-3
......................cgugauauugauucggcucauAu..............................................................................................................................................................................................   1     1   LT-2
......................cgugauauugauucggcucauUu..............................................................................................................................................................................................   2     1   LT-1
......................cgugauauugauucggcucaucA..............................................................................................................................................................................................   1     1   LCK-3
......................cgugauauugauucggcucauUu..............................................................................................................................................................................................   6     1   LT-2
......................cgugauauugauucggcucauUu..............................................................................................................................................................................................   9     1   LCK-3
......................cgugauauugCuucggcucaucu..............................................................................................................................................................................................   1     1   LT-1
......................cgugauauugauucggcucaucA..............................................................................................................................................................................................   3     1   LT-2
......................cgugauauugauucggcucaucu..............................................................................................................................................................................................   1     0   JCK-1
......................cgugauauugauucggcucaucu..............................................................................................................................................................................................   3     0   JCK-2
......................cAugauauugauucggcucaucu..............................................................................................................................................................................................   1     1   LT-2
......................cgugauauugauucggcucaucA..............................................................................................................................................................................................   3     1   LT-3
......................cgugauauugauucggcucauUu..............................................................................................................................................................................................   1     1   LT-3
......................cgugauauugauucggcucaucu..............................................................................................................................................................................................   17    0   LT-2
......................cgugauauugauucggcucaucu..............................................................................................................................................................................................   30    0   LT-1
......................cgugauauugauucggcucaucuU.............................................................................................................................................................................................   1     1   LT-2
......................cgugauauugauucggcucaucuU.............................................................................................................................................................................................   2     1   LT-3
......................cgugauauugauucggcucaucuU.............................................................................................................................................................................................   1     1   JCK-2
......................cgugauauugauucggcucaucuUu............................................................................................................................................................................................   1     1   LCK-3
.......................gugauauugauucggcuca.................................................................................................................................................................................................   1     0   LT-2
.......................gugauauugauucggcucauc...............................................................................................................................................................................................   1     0   LT-1
.......................gugauauugauucggcucauc...............................................................................................................................................................................................   2     0   LT-3
......................................................................................cgagccgaaucaauauca...................................................................................................................................................   1     0   LT-2
......................................................................................cgagccgaaucaauaucU...................................................................................................................................................   2     1   LCK-3
......................................................................................cgagccgaaucaauaucac..................................................................................................................................................   1     0   LT-1
......................................................................................cgagccgaaucaauaucacu.................................................................................................................................................   6     0   LT-1
......................................................................................cgagccgaaucaauaucacu.................................................................................................................................................   5     0   LT-2
......................................................................................cgagccgaaucaauaucacu.................................................................................................................................................   2     0   LT-3
......................................................................................cgagccgaaucaauaucacuU................................................................................................................................................   1     1   LT-1
```
